# Longitudinal peripheral blood immune profiling reveals dynamic changes of five major immune cell lineages and their clinical associations in pediatric scrub typhus

**DOI:** 10.3389/fimmu.2026.1904742

**Published:** 2026-09-14

**Authors:** Yuanyuan Hua, Yushen He, Jimin Zhou, Yuxiang Yang, Xingming Xu, Yan Zhang, Shuai Zhang, Xianqiu Luo, Jing Hu, Kaiyan Yue, Guolian Chen, Zuqun Yang, Rongju Wen, Feng Xu

**Affiliations:** 1 Department of Intensive Care Unit Children’s Hospital of Chongqing Medical University, National Clinical Research Center for Children and Adolescents’ Health and Diseases, Ministry of Education Key Laboratory of Child Development and Disorders, Chongqing Key Laboratory of Pediatric Metabolism and Inflammatory Diseases, Chongqing, China; 2 Department of Pediatrics, The First People’s Hospital of Liangshan Prefecture, Xichang, China; 3 College of Future Technology, Peking University, Beijing, China; 4 Department of Pediatrics, Jin Yang County People’s Hospital, Jinyang County, China; 5 Department of Clinical Laboratory, The First People’s Hospital of Liangshan Prefecture, Xichang, China; 6 Department of Pediatrics, Ning Nan County People’s Hospital, Ningnan County, China; 7 Department of Pediatrics, Hui Dong County People’s Hospital, Huidong County, China; 8 Department of Pediatrics, BuTuo County People’s Hospital, Butuo County, China; 9 Department of Pediatrics, Mian Ning County People’s Hospital, Mianning County, China

**Keywords:** children, disease severity, dynamic evolution, immune cell subsets, scrub typhus

## Abstract

**Objective:**

To investigate the dynamic changes of five major peripheral blood immune cell lineages during acute scrub typhus in children and their association with disease severity.

**Methods:**

A total of 141 children with clinically diagnosed scrub typhus were enrolled, including 100 mild and 41 severe cases. All children provided pretreatment immune samples, and 110 children with repeated post-treatment sampling contributed 299 observations to longitudinal immune analyses. Peripheral blood was collected at multiple time points, and the proportions and phenotypes of dendritic cells (DCs), monocytes, CD4^+^ T cells, CD8^+^ T cells, and B cells were analyzed by multicolor flow cytometry. Linear mixed-effects models were used to assess the dynamic changes of immune parameters over the disease course, and Spearman partial correlation analysis was performed to evaluate the associations between immune parameters and clinical indices.

**Results:**

Over the disease course, the proportion of HLA-DR^+^ monocytes increased most rapidly (FDR *q* = 0.003), and the proportions of DCs, CD4^+^ T cells, and CD8^+^ T cells also increased significantly (FDR *q* < 0.05). CD8^+^ T cells showed a reduction in naive subsets and an expansion of effector memory and TEMRA subsets. Among B cells, double-negative B cells decreased, while HLA-DR^+^ B cells and IgM memory B cells increased (FDR *q* < 0.05). Severe cases had significantly higher proportions of monocytes and B cells, but lower proportions of DCs, CD4^+^ T cells, and CD8^+^ T cells compared with mild cases (FDR *q* < 0.05). The proportion of activated CD8^+^ T cells was positively correlated with lactate dehydrogenase and D-dimer, and negatively correlated with platelet count (all FDR *q* < 0.001). After treatment, HLA-DR^+^ B cells and several monocyte subsets increased significantly (FDR *q* < 0.05).

**Conclusion:**

This study systematically delineates the dynamic evolution of five major immune cell lineages during acute scrub typhus in children, describing immune alterations including a rapid increase of HLA-DR^+^monocytes and a differentiation shift of CD8^+^ T cells toward an effector memory phenotype. It also demonstrates significant associations between activated CD8^+^ T cells and tissue injury as well as coagulation dysfunction. These findings provide novel longitudinal evidence for understanding the immunopathogenesis of scrub typhus in children.

## 1 Introduction

Scrub typhus is an important zoonotic disease caused by *Orientia tsutsugamushi*. In recent years, related new species such as *Candidatus Orientia chuto* have also been reported (1, 2). The disease has long been endemic in the Asia-Pacific region known as the “tsutsugamushi triangle”, but accumulating evidence indicates that its geographic distribution is gradually expanding beyond traditionally endemic areas (3, 4). Changes in temperature, humidity, and ecological conditions driven by climate change may affect the distribution of vector chigger mites and reservoir animal hosts, thereby serving as key factors driving this expansion trend and making scrub typhus an increasingly serious global public health threat (5, 6). A recent global systematic review and meta‑analysis showed that the seroprevalence of scrub typhus reached 10.73% in healthy populations and 22.58% in febrile patients, with a median case fatality rate of 5.00% among hospitalized patients (7).

The scrub typhus epidemic in China has spread from the southern and southwestern coastal regions to most provinces across the country (8). In Sichuan Province, the epidemic is highly concentrated in Liangshan Prefecture and Panzhihua City, which together account for 95.35% of the provincial cases. Among these, Liangshan Prefecture has an average annual incidence rate as high as 9.12/100,000, making it a clearly defined core endemic area (9). Unlike the situation in Shandong, Jiangsu, and Anhui provinces, where the elderly are the predominant affected population, Liangshan Prefecture has the highest incidence rate among children under 10 years of age (1.27/100,000), representing a prominent public health concern (10, 11). This epidemiological feature suggests that, in the scrub typhus endemic areas of southwestern China, children are the population with the highest infection risk and require the greatest attention.

The clinical management of scrub typhus in children faces significant challenges. On one hand, the clinical manifestations are highly atypical, and the detection rate of characteristic eschars varies greatly across regions and studies. Eschars are often located in concealed sites and can be easily missed, leading to frequent misdiagnosis as sepsis, hemophagocytic syndrome, or other conditions, resulting in a very high initial misdiagnosis rate (12). On the other hand, this disease shows a marked tendency toward severe illness in children, which can rapidly progress to multiple organ dysfunction involving the liver, kidneys, heart, lungs, and central nervous system, with a non-negligible case fatality rate (13).

Orientia tsutsugamushi is an obligate intracellular bacterium that primarily infects endothelial cells, monocytes/macrophages, and dendritic cells (DCs) (14). Regarding innate immunity, following infection, monocytes/macrophages are extensively activated and release large amounts of proinflammatory cytokines such as tumor necrosis factor-alpha (TNF-α) and interleukin-1β (IL-1β), while DCs are reduced in number and functionally impaired (15–17). In terms of adaptive immunity, both animal model and adult studies suggest that the infection induces a strong Th1-like CD8^+^ T cell response with significantly increased interferon-gamma (IFN-γ) secretion (15, 18). However, this T cell activation exhibits a “double-edged sword” characteristic — it is necessary for controlling the infection, but may also drive vascular endothelial injury and multiple organ dysfunction through the release of effector molecules (19). The role of B cells in scrub typhus has long been neglected, and only recently has a mouse model study revealed that infection can lead to disruption of splenic germinal center architecture and B cell dysfunction (20).

Of note, the above-mentioned immunological studies are almost all based on adult patients or animal models. The immune system of children is in the process of development and maturation, and the response intensity, regulatory mechanisms, and immune memory formation of both innate and adaptive immunity differ essentially from those in adults (21). Sepsis research has confirmed that children and adults exhibit distinct immune phenotypes (22). However, the systemic immune characteristics during the acute phase in children from highly endemic areas of scrub typhus remain a research gap. Specifically, the phenotypic profiles, dynamic evolution patterns, and associations with disease severity of dendritic cell subsets, monocyte subsets, CD4^+^ and CD8^+^ T cell differentiation subsets, and B cell subsets in pediatric acute scrub typhus have not been systematically studied. In particular, the dynamic evolution of immune cell subsets over the disease course, which is of great value for understanding disease progression and guiding clinical intervention, currently lacks description based on longitudinal data.

Against this background, what is already known and what remains unknown can be summarized as follows. It is well established that O. tsutsugamushi triggers robust activation of monocytes/macrophages and CD8^+^ T cells in adults and animal models, and that severe disease is associated with coagulation dysfunction and endothelial injury. However, whether these immune features are recapitulated in children—whose immune systems are developmentally distinct—has not been systematically examined. Moreover, the longitudinal dynamics of major immune lineages (T cells, B cells, monocytes, and dendritic cells) during the acute phase of pediatric scrub typhus have not been described, nor have their associations with disease severity been quantitatively assessed in children.

To address these gaps, this study was designed to: (1) systematically characterize the dynamic changes of five major immune cell lineages—CD4^+^ and CD8^+^ T cells (including their differentiation and activation subsets), B cells (including memory and regulatory subsets), monocytes (including classical, intermediate, and non-classical subsets), and dendritic cells (including cDC1 and pDC subsets)—in children with acute scrub typhus; (2) compare the immune profiles between mild and severe cases to identify severity-associated immune features; and (3) explore the associations between immune parameters and clinical indicators of disease severity, including coagulation dysfunction, liver injury, and inflammation. By providing the first longitudinal immune profiling data in pediatric scrub typhus, this study aims to generate hypotheses about the immunopathological mechanisms underlying severe disease in children and to inform future mechanistic and biomarker studies.

## 2 Materials and methods

### 2.1 Diagnostic criteria

Diagnosis was made according to the Expert Consensus on the Clinical Diagnosis and Treatment of Scrub Typhus (2024) (23). A clinical diagnosis was established if any of the following criteria were met: (1) history of epidemiological exposure accompanied by typical clinical manifestations (eschar, rash, lymphadenopathy); (2) positive scrub typhus-specific IgM antibody, a fourfold or greater rise in serum IgG antibody titer during convalescence, or a positive PCR result; (3) highly suspected cases showing defervescence within 48 hours after empirical treatment with doxycycline or azithromycin. Routine IgM/IgG antibody testing was not performed due to limitations in laboratory resources at the participating county-level hospitals. Therefore, all cases in this cohort are clinically diagnosed according to the consensus criteria.

### 2.2 Inclusion and exclusion criteria

Inclusion criteria: (1) meeting the diagnostic criteria above; (2) age≥28 days and < 18 years; (3) written informed consent provided by a legal guardian.

Exclusion criteria:coexisting other definite acute infections, primary immunodeficiency, hematological disorders, liver diseases, or long-term use of immunosuppressive agents.

### 2.3 Definition of severe group

Patients were classified as severe cases according to the criteria proposed by Park (24), if any of the following was present: central nervous system involvement (altered consciousness, seizures, cerebral hemorrhage or infarction), respiratory involvement (bilateral pulmonary infiltrates with an oxygenation index ≤ 250 mmHg or requirement for mechanical ventilation), myocarditis/myocardial ischemia/new-onset arrhythmia, renal impairment (serum creatinine≥176μmol/L), septic shock, gastrointestinal bleeding, or death. The remaining children were assigned to the mild group.

### 2.4 Clinical data collection

Demographic data, epidemiological history, symptoms and signs, complications, laboratory findings, and treatment outcomes were collected using a standardized case report form. Laboratory parameters included complete blood count, C-reactive protein, liver function, renal function, coagulation profile, and electrolytes.

### 2.5 Flow cytometry

Peripheral venous blood (1mL) was collected from enrolled children on the day of admission (before treatment) and at multiple follow-up time points into EDTA-anticoagulated tubes. Samples were transported to the laboratory at 2–8 °C and processed immediately. The first day of fever was designated as day 0, and all sampling time points were expressed as days after fever onset. Immunophenotyping was performed on a Cytek Aurora multispectral flow cytometer (Cytek Biosciences, USA).

For each sample, 100 μL of EDTA-anticoagulated whole blood was mixed with 1 mL of red blood cell lysis buffer, incubated at room temperature for 10 min, and centrifuged at 550 × g for 5 min. After discarding the supernatant, the cell pellet was resuspended in 200 μL of FACS buffer (PBS + 2% FBS). Seventy-five microliters of BD Horizon Brilliant Stain Buffer was added, followed by the addition of fluorophore-conjugated antibodies against CD8, CD4, KLRG1, CD45RA, CD86, CD14, CD16, CD141, CD123, HLA-DR, CD11c, CD45, CD3, IgD, CD27, CD19, CD38, and CD20, along with a Live/Dead fixable viability dye, at the manufacturer’s recommended amounts (clone, fluorophore, and catalog number details are provided in the **Supplementary Table 1**). The mixture was vortexed and incubated at 4 °C in the dark for 30 min. Cells were washed by adding 1 mL of FACS buffer, centrifuging at 550 × g for 5 min, and discarding the supernatant; this wash step was repeated once. The stained cells were finally resuspended in 500 μL of FACS buffer, filtered, and acquired on the flow cytometer.

The gating strategy was sequentially applied as follows: single cells were first identified by FSC-A/FSC-H and SSC-A/SSC-H, followed by selection of live cells based on Live/Dead dye negativity, and then CD45^+^ leukocytes were gated. CD3^+^ T cells were divided into CD4^+^ and CD8^+^ subsets. For each subset, differentiation status was determined by CD27/CD45RA expression (naive: CD27^+^CD45RA^+^; central memory: CD27^+^CD45RA^–^; effector memory: CD27^–^CD45RA^–^; TEMRA: CD27^–^CD45RA^+^), activation level was assessed by HLA-DR^+^CD38^+^ co-expression, and KLRG1 expression was measured. CD3^–^CD19^+^ B cells were classified into four subsets based on CD27 and IgD expression (naive: IgD^+^CD27^–^; IgM memory: IgD^+^CD27^+^; switched memory: IgD^–^CD27^+^; double-negative: IgD^–^CD27^–^), and HLA-DR expression was also evaluated. Within the CD3^–^CD19^–^ cell population, CD14 and CD16 were used to distinguish three monocyte subsets (classical: CD14^+^CD16^–^; intermediate: CD14^+^CD16^+^; non-classical: CD14^–^CD16^+^), and HLA-DR expression was assessed. Dendritic cells (DCs) were defined as CD3^–^CD19^–^CD14^–^CD16^–^HLA-DR^+^ cells, which were further subdivided into cDC1 (CD11c^+^CD141^+^) and pDC (CD123^+^) subsets, and CD86 expression was measured as an activation marker. Proportions of all subsets are expressed as a percentage of their respective parent gate. A representative gating strategy is shown in **Supplementary Figure 1**.

Spectral unmixing for the Cytek Aurora system was performed using the instrument’s built-in automatic unmixing algorithm with single-stained controls. Fluorescence-minus-one (FMO) controls and batch-correction procedures were not performed in this study. Samples were acquired over multiple experimental sessions; to minimize inter-run variability, all sessions used the same lot of antibodies and identical instrument settings, and a standardized gating template was applied to all samples. Operators were blinded to the clinical severity group throughout sample acquisition and data analysis. Raw data were analyzed using FlowJo software (Tristar, San Carlos, CA, USA).

### 2.6 Study cohorts and sampling structure

A total of 141 children with confirmed scrub typhus were enrolled, including 100 mild and 41 severe cases. All children provided pretreatment baseline immune samples. Among them, 110 children underwent repeated post-treatment sampling and contributed 299 observations to longitudinal immune analyses, with 2–4 immune measurements per child (median: 3). Sampling frequency was virtually identical between severity groups: the severe group (33 children, 89 observations) and the mild group (77 children, 210 observations) both had a median of 3 samples per child (range: 2–4), with no statistically significant difference between groups (P = 0.571, Mann-Whitney U test). The remaining 31 children had only pretreatment samples because their guardians declined subsequent blood sampling; they remained included in the clinical and baseline immune analyses but were excluded from longitudinal mixed-effects analyses. Their severity distribution and baseline clinical characteristics were comparable to those of the 110 children included in the longitudinal immune cohort, as shown in **Supplementary Table 2**.

### 2.7 Statistical analysis

All analyses were performed using R version 4.5.2. Continuous variables were expressed as mean ± SD or median (IQR) depending on normality, and compared using the t-test or Mann-Whitney U test. Categorical variables were presented as frequency (%) and compared by the χ^2^ test or Fisher ‘s exact test. Multiple comparisons were corrected using the Benjamini-Hochberg procedure to control the false discovery rate (FDR), with an FDR q < 0.05 considered significant.

Dynamic changes in immune markers were analyzed with linear mixed-effects models, including patient as a random intercept, and fixed effects of days after fever onset, group, and their interaction, with age and sex additionally included as fixed effects to adjust for potential confounding. To assess the robustness of the findings, sensitivity analyses were performed by including age and sex as covariates in the models; the results of these adjusted models are presented in **Supplementary Tables 3a–c**. The optimal random-effects structure (random intercept only vs. random intercept with random slope for time) was selected based on likelihood ratio tests. A separate treatment-effect model was fitted, with treatment status, treatment days, group, and their interactions as fixed effects. All P values from the mixed-effects models were FDR-adjusted.

Model assumptions were verified by residual diagnostics. Residual plots and Q-Q plots showed approximately normally distributed residuals with no substantial heteroscedasticity (**Supplementary Figure 3**), supporting the use of linear mixed-effects models on the original percentage scale. Sensitivity analyses using logit-transformed proportions yielded results consistent with the primary analysis.

For immune-clinical association analyses using Spearman partial correlation, only baseline (pre-treatment) measurements were used to ensure independence of observations; repeated post-treatment measurements from the same patient were not included. Age was included as a covariate in these correlation analyses, and P values were FDR-adjusted.

A total of 141 children were enrolled, of whom 110 with ≥2 immune measurements (299 observations) contributed to longitudinal analyses. The days after fever onset at admission did not differ significantly between groups (P = 0.135), confirming baseline comparability.

## 3 Results

### 3.1 Clinical characteristics

A total of 141 children with confirmed scrub typhus were enrolled, including 100 mild cases and 41 severe cases. The 31 children with baseline-only immune samples did not differ significantly from the 110 children included in the longitudinal immune cohort in severity distribution or baseline clinical characteristics (**Supplementary Table 2**). All patients had a definite history of outdoor activity and characteristic eschars (most common locations: axilla 14.2%, scrotum 13.5%, groin 9.9%). Compared with the mild group, the severe group had significantly higher incidences of cough(*P* < 0.001), hepatosplenomegaly(*P* < 0.001), and rash (*P* = 0.028), but a lower incidence of headache (*P* = 0.017). No significant differences were observed in abdominal pain, vomiting, or lymphadenopathy between the two groups. Regarding complications, the severe group had significantly higher rates of disseminated intravascular coagulation (85.4%), hypoalbuminemia (80.5%), severe liver dysfunction (65.9%), pneumonia (68.3%), hypofibrinogenemia (70.7%), thrombocytopenia (56.1%), and hyponatremia (68.3%) (all *P* < 0.05) (**Table 1**).

**Table 1:** Comparison of clinical characteristics, complications, and therapeutic drugs between the two groups [n(%)].

	All (N=141)	Severe group (N=41)	Mild group (N=100)	χ^2^/Fisher	*P*
Sex				1.228	0.268
male	88 (62.4)	23 (56.1)	65 (65.0)		
Female	53 (37.6)	18 (43.9)	35 (35.0)		
Outdoor activity	141 (100)	41 (100)	100 (100)	–	–
Manifestation					
Fever	141 (100)	41 (100)	100 (100)	–	–
Abdominal pain	13 (9.2)	6 (14.6)	7 (7.0)	Fisher	0.207
Vomiting	10 (7.1)	3 (7.3)	7 (7.0)	Fisher	1.000
Headache	49 (34.8)	8 (19.5)	41 (41.0)	5.916	0.015
Cough	53 (37.6)	25 (61.0)	28 (28.0)	13.430	<0.001
Physical signs					
Eschar	141 (100)	41 (100)	100 (100)	–	–
Rash	5 (3.5)	4 (9.8)	1 (1.0)	Fisher	0.024
Lymphadenopathy	62 (44.0)	19 (46.3)	43 (43.0)	0.131	0.717
Hepatosplenomegaly	17 (12.1)	14 (34.1)	3 (3.0)	Fisher	<0.001
Complication					
Liver damage	34 (24.1)	27 (65.9)	7 (7.0)	Fisher	<0.001
Hypoalbuminemia	39 (27.7)	33 (80.5)	6 (6.0)	Fisher	<0.001
Hypofibrinogenemia	37 (26.2)	29 (70.7)	8 (8.0)	Fisher	<0.001
DIC	36 (25.5)	35 (85.4)	1 (1.0)	Fisher	<0.001
Pneumonia	38 (27.0)	28 (68.3)	10 (10.0)	Fisher	<0.001
Hyponatremia	75 (53.2)	28 (68.3)	47 (47.0)	5.324	0.021
Thrombocytopenia	25 (17.7)	23 (56.1)	2 (2.0)	Fisher	<0.001
Doxycycline	96 (68%)	18 (44%)	78 (78%)		
Azithromycin	45 (32%)	23 (56%)	22 (22%)	Fisher	<0.001

After Benjamini-Hochberg correction, 16 of the 18 laboratory parameters showed significant differences between the two groups (FDR *q* < 0.05), except for neutrophil and lymphocyte percentages. The severe group had significantly longer fever duration and hospital stay (FDR *q* < 0.001). Levels of platelets, hemoglobin, albumin, serum sodium, and fibrinogen were significantly lower in the severe group, whereas white blood cell count, D-dimer, activated partial thromboplastin time, prothrombin time, lactate dehydrogenase, aspartate aminotransferase, alanine aminotransferase, and C-reactive protein were significantly higher (FDR *q* < 0.01). The median age of the severe group was significantly younger than that of the mild group (FDR *q* = 0.004) (**Table 2**).

**Table 2:** Comparison of clinical course and laboratory parameters between the two groups [M (Q1, Q3)].

Variable	Mild group (N=100)	Severe group (N = 41)	Z	FDR *P*
Age (m)	108.00 (60.00-144.00)	72.00 (27.00-108.00)	3.055	0.004
Days after fever onset (d)	5.00 (3.00-7.00)	4.00 (2.00-6.00)	1.493	—
Fever duration (d)	7.00 (5.00-8.00)	9.00 (7.00-10.00)	-4.413	<0.001
Hospital stay (d)	7.00 (6.00-9.00)	12.00 (10.00-14.00)	-7.291	<0.001
WBC (×10^9^/L)	5.61 (3.86-7.69)	7.83 (4.94-10.28)	-3.028	0.004
Hb (g/L)	118.50 (110.00-132.25)	108.00 (94.00-114.00)	4.797	<0.001
PLT (×10^9^/L)	172.50 (115.50-233.00)	42.00 (31.00-53.00)	8.253	<0.001
CRP (mg/L)	33.98 (14.17-60.03)	110.43 (72.32-158.65)	-6.815	<0.001
N (%)	67.05 (53.38-76.15)	65.10 (50.00-73.90)	0.806	0.456
L (%)	24.75 (17.35-36.08)	25.00 (18.40-43.75)	-0.676	0.523
ALT (U/L)	31.17 (17.24-61.45)	193.73 (110.30-278.96)	-8.175	<0.001
AST (U/L)	49.17 (32.50-82.31)	253.00 (192.53-446.85)	-8.737	<0.001
ALB (g/L)	39.83 (35.64-43.88)	25.40 (22.76-28.34)	8.422	<0.001
LDH (U/L)	403.00 (267.18-519.30)	819.2 (731.50-1012.20)	-7.877	<0.001
PT (s)	16.20 (14.05-18.68)	21.10 (17.90-24.60)	-5.791	<0.001
APTT (s)	31.55 (28.52-36.40)	42.80 (38.40-47.85)	-6.964	<0.001
Fib (g/L)	2.39 (2.02-2.70)	1.04 (0.69-1.22)	8.203	<0.001
D-dimer (mg/L)	2.78 (1.20-5.22)	16.06 (8.70-19.29)	-7.863	<0.001
Na^+^ (mmol/L)	134.55 (131.78-137.00)	129.20 (126.85-132.40)	4.976	<0.001

### 3.2 Associations between immune cell subsets and clinical parameters

Spearman partial correlation analysis revealed associations between multiple immune cell subsets and parameters of coagulation, liver injury, and inflammation: HLA-DR^+^ DC proportion was positively correlated with D-dimer (r = 0.321, FDR *q* = 0.004) and ALT (r = 0.327, FDR *q* = 0.004); HLA-DR^+^ monocyte proportion was negatively correlated with fibrinogen (r = -0.367, FDR *q* < 0.001); resting CD4^+^ T cell proportion was positively correlated with LDH (r = 0.506, FDR *q* < 0.001) and negatively correlated with platelet count (r = -0.434, FDR *q* < 0.001); activated CD8^+^ T cell proportion was positively correlated with LDH (r = 0.495, FDR *q* < 0.001) and D-dimer (r = 0.443, FDR *q* < 0.001), and negatively correlated with platelet count (r = -0.378, FDR *q* < 0.001) and albumin (r = -0.427, FDR *q* < 0.001); plasmablast proportion was positively correlated with LDH (r = 0.477, FDR *q* < 0.001) and D-dimer (r = 0.405, FDR *q* < 0.001), and negatively correlated with platelet count (r = -0.290, FDR *q* = 0.009) (**Table 3**).

**Table 3:** Spearman partial correlation between immune cell subsets and clinical parameters (adjusted for age).

Cell subset	Clinical parameter	r	FDR *q*
HLA-DR^+^ DC	D-dimer	0.321	0.004
HLA-DR^+^ DC	ALT	0.327	0.004
HLA-DR^+^ monocyte	Fibrinogen	-0.367	<0.001
Resting CD4^+^ T	LDH	0.506	<0.001
Resting CD4^+^ T	Platelet count	-0.434	<0.001
Activated CD8^+^ T	LDH	0.495	<0.001
Activated CD8^+^ T	D-dimer	0.443	<0.001
Activated CD8^+^ T	Platelet count	-0.378	<0.001
Activated CD8^+^ T	Albumin	-0.427	<0.001
Plasmablast	LDH	0.477	<0.001
Plasmablast	D-dimer	0.405	<0.001
Plasmablast	Platelet count	-0.290	0.009

### 3.3 Dynamic evolution of immune cell subsets

#### 3.3.1 Overall dynamic trends

Linear mixed-effects model analysis revealed that the proportions of several immune cell subsets changed significantly over the disease course (time effect, FDR *q* < 0.05). The proportion of HLA-DR^+^ monocytes showed the most rapid increase (β = 2.77, FDR *q* = 0.003). The proportions of DCs (β = 1.79, FDR *q* = 0.042), CD4^+^ T cells (β = 1.43, FDR *q* = 0.010), and CD8^+^ T cells (β = 1.27, FDR *q* = 0.015) also increased significantly. Double-negative B cells decreased over time (β = –0.89, FDR *q* = 0.021), while HLA-DR^+^ B cells (β = 2.21, FDR *q* < 0.001) and IgM memory B cells (β = 1.31, FDR *q* = 0.031) increased. Among CD8^+^ T cell subsets, central memory (β = 1.58, FDR *q* = 0.015) and terminally differentiated effector memory cells (β = 0.62, FDR *q* = 0.040) also increased significantly (**Table 4**). To visualize these temporal dynamics, we plotted the model-predicted trajectories of the five major immune lineages over the disease course (**Figure 1**).

**Table 4:** Time effects of immune cell subsets during disease course.

Immune cell subset	β	FDR *q*
HLA-DR^+^ monocytes	2.77	0.003
Total DCs	1.79	0.042
Total CD4^+^ T cells	1.43	0.010
Total CD8^+^ T cells	1.27	0.015
Central memory CD8^+^ T cells	1.58	0.015
TEMRA CD8^+^ T cells	0.62	0.040
HLA-DR^+^ B cells	2.21	<0.001
IgM memory B cells	1.31	0.031
Double-negative B cells	–0.89	0.021

**Figure 1:**
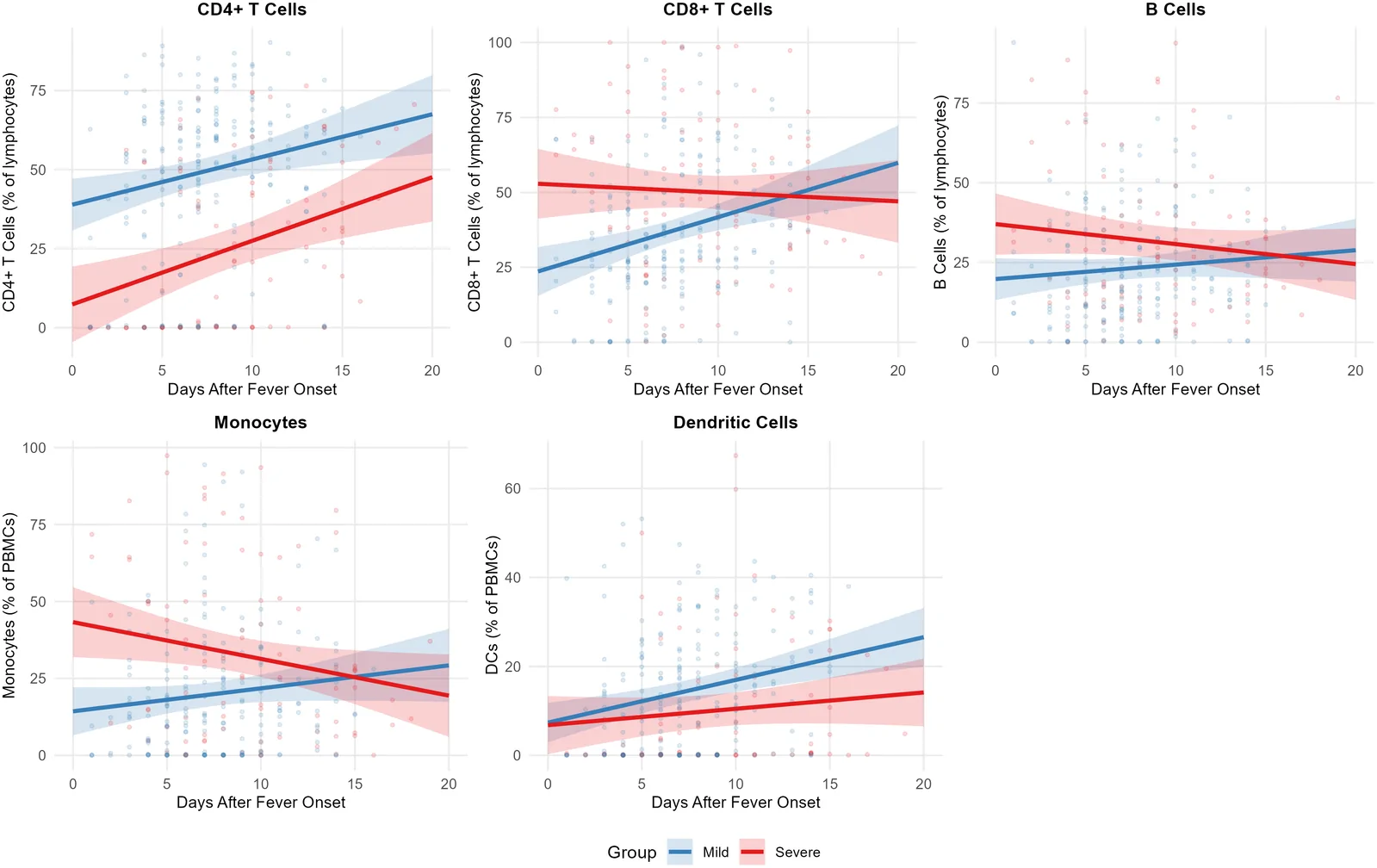
Longitudinal trajectories of five major immune cell lineages over the disease course. Lines represent model-predicted trends from linear mixed-effects models for each major lineage (total CD4^+^ T cells, total CD8^+^ T cells, total B cells, total monocytes, and total dendritic cells), stratified by disease severity (severe: red; mild: blue). Shaded areas indicate 95% confidence intervals. The x-axis represents days after fever onset; the y-axis represents the percentage of each subset.

To evaluate the potential confounding effects of age and sex, we included these two variables as covariates in the mixed-effects models as a sensitivity analysis. After adjustment, the temporal increases in HLA-DR^+^ B cells (adjusted β = 1.67, FDR q = 0.026) and total CD4^+^ T cells (adjusted β = 0.020, FDR q < 0.001) remained significant, suggesting robust time effects for these subsets. However, the time effects of IgM memory B cells, double-negative B cells, total CD8^+^ T cells, central memory CD8^+^ T cells, TEMRA CD8^+^ T cells, total DCs, and HLA-DR^+^ monocytes were no longer significant after adjustment (**Supplementary Table 3a**), indicating varying degrees of confounding by age and sex for these subsets.

#### 3.3.2 Differences between mild and severe group

Severe cases had significantly higher proportions of total monocytes (β = 28.96, FDR *q* = 0.001) and total B cells (β = 17.22, FDR*q* = 0.021), but significantly lower proportions of total DCs (β = 20.37, FDR *q* = 0.042), CD4^+^ T cells (β = –31.53, FDR *q* < 0.001), and CD8^+^ T cells (β = –28.76, FDR *q* < 0.001) compared with mild cases. A significant time × group interaction was observed for total monocyte proportion (β = –1.94, FDR *q* = 0.029), indicating a slower rate of increase in severe cases. No significant interactions were found for other subsets, suggesting largely parallel trajectories between the two groups (**Table 5**).

**Table 5:** Group effects of immune cell subsets between severe and mild group.

Immune cell subset	β	FDR *q*
Total monocytes	28.96	0.001
Total DCs	-20.37	0.042
Total CD4^+^ T cells	–31.53	<0.001
Total CD8^+^ T cells	–28.76	<0.001
Total B cells	17.22	0.021
Naive B cells	20.12	0.021
Switched memory B cells	−12.14	0.021

β coefficients represent the difference between severe and mild groups (reference: severe group). Positive β indicates higher values in severe cases; negative β indicates lower values in severe cases.

In sensitivity analyses adjusting for age and sex, the group differences between severe and mild cases remained significant and directionally consistent for B cell percentage (adjusted β = −15.98, FDR q = 0.007), naive B cells (adjusted β = 19.14, FDR q = 0.006), switched memory B cells (adjusted β = −11.42, FDR q = 0.007), total CD4^+^ T cells (adjusted β = 0.328, FDR q < 0.001), and total CD8^+^ T cells (adjusted β = −29.98, FDR q < 0.001) (**Supplementary Table 3b**). In contrast, the group difference for total DCs was no longer significant after adjustment (adjusted β = 1.04, FDR q = 0.798), and the direction of the group difference for total monocytes reversed after adjustment (adjusted β = −28.98, FDR q < 0.001), suggesting strong confounding by age and sex for these two lineages.

#### 3.3.3 Treatment effects

After treatment, HLA-DR^+^ B cells increased significantly (β = 14.83, FDR *q* = 0.035). Total monocytes (β = 11.32, FDR *q* = 0.018), classical monocytes (β = 20.36, FDR *q* = 0.011), and HLA-DR^+^ monocytes (β = 17.87, FDR *q* = 0.030) also increased significantly compared with baseline. Treatment duration had a positive effect on CD86 expression on DCs (β = 1.09, FDR *q* = 0.017). No significant changes were observed for other subsets after treatment (**Table 6**).

**Table 6:** Immune cell subsets before and after treatment.

Immune cell subset	β	FDR *q*
Total monocytes	11.32	0.018
Classical monocytes	20.36	0.011
HLA-DR^+^ monocytes	17.87	0.030
HLA-DR^+^ B cells	14.83	0.035

## 4 Discussion

This study provides a systematic description of the dynamic evolution of five major immune cell lineages during acute scrub typhus in children and their association with disease severity. Clinical data showed that severe cases were characterized by coagulation-fibrinolysis dysfunction, increased vascular permeability, and multi-organ injury, with a DIC incidence of 85.4%, decreased fibrinogen, elevated D-dimer, and thrombocytopenia, consistent with adult severe scrub typhus (25–27), suggesting widespread vascular endothelial injury as a potential core pathological basis. Monocytes infected by Orientia tsutsugamushi can secrete inflammatory cytokines that induce endothelial activation (28), and population studies have demonstrated a positive correlation between coagulation activation and pro-inflammatory responses (26, 29).

At the innate immunity level, the proportion of HLA-DR^+^ monocytes increased rapidly over the disease course (β = 2.77/day, FDR *q* = 0.003), representing the most rapidly rising immune indicator, suggesting sustained monocyte activation. This finding is consistent with *in vitro* experiments and eschar histopathology (28, 30, 31). Notably, both mild and severe cases showed a significant increase, with only a higher baseline in severe cases. This suggests that sustained monocyte activation is a universal innate immune response during acute scrub typhus rather than a severe-specific immune deviation; the degree of baseline activation may determine disease severity, and the admission level of monocyte activation could serve as a potential early marker for identifying severe disease.

After treatment, the proportions of total monocytes, classical monocytes, and HLA-DR^+^ monocytes all increased significantly compared with pre-treatment values, and severe cases still had higher levels than mild cases. This phenomenon requires careful interpretation. One possible interpretation is that effective anti-infective treatment curtails the systemic inflammatory storm (32, 33); allowing monocytes to be released from persistent activation, with HLA-DR up-regulation potentially reflecting resolution of immunoparalysis. Alternatively, tissue-infiltrating monocytes may re-enter the peripheral circulation after inflammation resolves (34, 35). A third possible mechanism—compensatory emergency myelopoiesis enhancing monocyte production (36, 37)– is not well supported by our data, as other myeloid lineages (e.g., neutrophils) did not show a parallel increase. However, distinguishing among these interpretations is not possible with our current data; functional assays and longitudinal assessments of antigen-presenting capacity are needed to determine whether HLA-DR up-regulation represents genuine immune recovery, persistent activation, or ongoing inflammation. Whether the persistently higher monocyte levels in severe cases after treatment represent ‘residual activation’ or ‘compensatory overproduction’ needs to be clarified by functional assays and long-term follow-up.

At the adaptive immunity level, severe cases showed a differentiation shift of CD8^+^ T cells toward effector memory and TEMRA phenotypes, with decreased naive subsets and increased KLRG1^+^ senescent/terminally differentiated cells, consistent with a mouse model (19). Children’s CD8^+^ T cells naturally possess resistance to immune exhaustion (38); the elevated KLRG1 expression in severe cases suggests that persistent and intense antigen stimulation may disrupt the inherent immune differentiation pattern of children, potentially contributing to premature T-cell senescence/terminal differentiation, which may be associated with aggravated immunopathological injury in severe disease.

More importantly, activated CD8^+^ T cells were positively correlated with LDH (r = 0.495) and D-dimer (r = 0.443), and negatively correlated with platelet count (r = –0.378) and albumin (r = –0.427) (all FDR q < 0.001), providing correlative evidence at the T-cell level suggestive of “immune-coagulation crosstalk”. Although previous studies have linked coagulation activation to inflammatory cytokines, the present study extends this chain to CD8^+^ T cells, raising the possibility that activated CD8^+^ T cells may may play a role in connecting immune activation and vascular endothelial injury. We hypothesize that they damage the endothelium directly or indirectly (e.g., through IFN-γ, granzyme B) and trigger the coagulation cascade. Although previous studies have linked coagulation activation to inflammatory cytokines (29), the present study extends this chain to CD8^+^ T cells, clearly suggesting that activated CD8^+^ T cells may play a role in connecting immune activation and vascular endothelial injury. We hypothesize that they could contribute to the endothelium directly or indirectly (e.g., through IFN-γ, granzyme B) and potentially contribute to the coagulation cascade, but this hypothesis requires functional validation in future studies. Notably, a recent study using multiparametric flow cytometry in children with multisystem inflammatory syndrome (MIS-C) also reported T-cell polarization toward IL-17 and IFN-γ production during severe inflammatory states, supporting the notion that pediatric T-cell activation may represent a shared immune feature across different severe inflammatory conditions in children (39).

In sharp contrast, the associations of CD4^+^ T-cell subsets with clinical injury markers were much weaker, and their dynamic changes were small. This difference raises the possibility that CD8^+^ T cells may be more prominently involved than CD4^+^ T cells in the immunopathological process during acute severe scrub typhus in children, which is consistent with murine studies showing that CD8^+^ T-cell depletion alleviates liver and lung injury while CD4^+^ T-cell depletion does not confer such protection (19). However, we acknowledge that immunopathological injury in scrub typhus is a multifaceted process involving innate immune cells (monocytes/macrophages, DCs), the coagulation cascade, endothelial dysfunction, and other adaptive immune components; the relative contribution of each subset warrants further investigation.

Regarding B cells, severe cases exhibited reduced memory B cells and increased double-negative B cells, which may reflect delayed humoral immune responses and impaired memory formation. Although plasmablast proportions were positively correlated with LDH and D-dimer and negatively correlated with platelet count, consistent with B-cell activation parallels systemic inflammation (39), the magnitude of dynamic changes was small, raising the possibility that B-cell activation may occur downstream of systemic inflammation rather than a driver of acute injury. Similar patterns of plasmablast activation have been observed in other acute infections, such as dengue (41).

The overall proportion of DCs increased only slightly, was higher in mild cases than in severe cases, and showed no significant improvement after treatment. This finding may deserve attention, as DCs are a key hub bridging innate and adaptive immunity, and delayed recovery of DC numbers could potentially affect subsequent immune responses. The short observation period (median hospital stay 12 days) is much shorter than the time required for full DC recovery (approximately 28 days), which may be the main reason (40). This raises the possibility that even after clinical symptoms improve, full immune recovery may take considerably longer, and post-discharge immune monitoring could be worth considering in future studies.

## 5 Conclusion

In summary, severe pediatric scrub typhus is characterized by widespread vascular endothelial injury as the core pathological basis, with immune features of sustained monocyte activation and a differentiation shift of CD8^+^ T cells toward effector memory/senescent phenotypes. Activated CD8^+^ T cells are strongly associated with coagulation dysfunction and tissue injury. This study provides the first systematic dynamic map of five major immune cell lineages during acute scrub typhus in children, HLA-DR^+^ monocytes and activated CD8^+^ T cells were found to be associated with disease severity, and correlative evidence at the T-cell level suggests a potential link between immune activation and coagulation dysfunction. However, these findings are hypothesis-generating; causality and mechanisms require functional validation in future studies. Whether immunomodulatory strategies could have a role in severe scrub typhus warrants further investigation in preclinical models and controlled clinical studies.

## 6 Limitation

Limitations include the single-center observational design, limited sample size, lack of functional assays, and short follow-up period, and the absence of healthy controls or febrile disease controls, which precludes any inference of disease specificity from the present data. These findings warrant validation in multicenter external cohorts. We also acknowledge that the longitudinal percentage changes may partially reflect denominator effects and should be interpreted with caution. Furthermore, we did not measure endothelial activation markers, effector molecules (IFN-γ, granzyme B, perforin), or cytokine secretion profiles; therefore, the proposed immune-coagulation link remains correlative and hypothesis-generating, requiring functional validation in future studies.

## Data Availability

The original contributions presented in the study are included in the article/**Supplementary Material**. Further inquiries can be directed to the corresponding author.
