# Supplementary material for: Longitudinal peripheral blood immune profiling reveals dynamic changes of five major immune cell lineages and their clinical associations in pediatric scrub typhus
Source: Front Immunol. 2026 Sep 14;17:1904742. doi: 10.3389/fimmu.2026.1904742 (PMC13616781; doi:10.3389/fimmu.2026.1904742)
Supplement: Supplementary file 1 [file SupplementaryFile1.docx]

**Appendix**

**Table S1 Antibodies used for flow cytometry**

| Antibody | Fluorophore | Clone | Catalog # | Brand |
| --- | --- | --- | --- | --- |
| CD8 | ALEX FLUOR700 | RPA-T8 | 561453 | BD Biosciences |
| CD4 | RB780 | RPA-T4 | 755704 | BD Biosciences |
| KLRG1 | RB613 | Z7-205.rMAb | 571358 | BD Biosciences |
| CD45RA | BUV395 | HI100 | 568712 | BD Biosciences |
| CD86 | BUV737 | 24F | 749219 | BD Biosciences |
| CD14 | BV605 | M5E2 | 564055 | BD Biosciences |
| CD16 | BUV615 | B73.1 | 751323 | BD Biosciences |
| CD141 | APC | 1A4 | 564123 | BD Biosciences |
| CD123 | RY610 | 7G3 | 759101 | BD Biosciences |
| HLA-DR | RB705 | L243 | 572764 | BD Biosciences |
| CD11C | FITC | B-ly6 | 561355 | BD Biosciences |
| L/D | FVS780 | — | — | Invitrogen |
| CD45 | BUV805 | HI30 | 612891 | BD Biosciences |
| CD3 | PACIFIC BLUE | SK7 | 563798 | BD Biosciences |
| IgD | BV510 | IA6-2 | 563110 | BD Biosciences |
| CD27 | PE-CY7 | O323 | 567289 | BD Biosciences |
| CD19 | PE-CY5 | HIB19 | 560993 | BD Biosciences |
| CD38 | BV786 | HIT2 | 563964 | BD Biosciences |
| CD20 | BV421 | 2H7 | 562873 | BD Biosciences |

Figure S1 Flow loop gate strategy

Single cells, live cells, and CD45⁺ leukocytes were sequentially gated. CD3⁺ T cells were divided into CD4⁺ and CD8⁺ subsets. For each subset, differentiation status was determined by CD27/CD45RA expression, activation level was assessed by HLA‑DR/CD38 co‑expression, and KLRG1 expression was measured. CD3⁻CD19⁺ B cells were classified into four subsets based on CD27 and IgD expression, and HLA‑DR expression was also evaluated. Within the CD3⁻CD19⁻ cell population, CD14 and CD16 were used to distinguish three monocyte subsets, and HLA‑DR expression was assessed. Dendritic cells (DCs) were defined as CD3⁻CD19⁻CD14⁻CD16⁻HLA‑DR⁺ cells, which were further subdivided into cDC1 and pDC subsets, and CD86 expression was measured as an activation marker. Proportions of all subsets are expressed as a percentage of their respective parent gate.

Supplementary Table S2. Baseline characteristics of the longitudinal immune cohort and baseline-only cohort

| Characteristic | Longitudinal cohort (n=110) | Baseline-only  (n=31) | *P* |
| --- | --- | --- | --- |
| Age (m) | 108.0 (60.0–144.0) | 96.0 (48.0–144.0) | 0.573 |
| Male (%) | 66 (60.0) | 22 (71.0) | 0.256 |
| Severe cases (%) | 33 (30.0) | 8 (25.8) | 0.641 |
| Days after fever onset (d) | 5.0 (3.0–7.0) | 4.0 (2.0–6.0) | 0.134 |
| WBC（×10⁹/L） | 6.18 (3.98–8.32) | 6.42 (3.75–9.03) | 0.827 |
| Hb（g/L） | 117.0 (106.0–128.0) | 116.0 (104.0–127.0) | 0.755 |
| Platelet count（×10⁹/L） | 134.0 (73.0–215.0) | 134.0 (61.0–199.0) | 0.677 |
| CRP（mg/L） | 43.6 (14.2–80.2) | 58.9 (16.3–99.7) | 0.423 |
| ALT（U/L） | 39.0 (18.0–95.0) | 62.3 (23.8–192.4) | 0.330 |
| AST（U/L） | 58.8 (34.1–128.0) | 74.4 (44.1–195.9) | 0.372 |
| ALB（g/L） | 39.1 (32.8–43.8) | 38.7 (33.5–43.1) | 0.692 |
| LDH（U/L） | 465.2 (329.0–579.3) | 490.3 (348.9–622.7) | 0.617 |
| PT（S） | 16.6 (14.6–19.9) | 16.6 (14.9–18.9) | 0.926 |
| APTT（S） | 32.4 (28.5–38.0) | 32.8 (29.5–38.0) | 0.883 |
| Fib(g/L) | 2.38 (1.63–2.73) | 2.31 (1.60–2.64) | 0.591 |
| D-dimer(mg/L) | 3.05 (1.17–6.35) | 4.06 (1.45–8.78) | 0.426 |
| Sodium(mmol/L) | 134.6 (131.0–137.0) | 134.5 (131.6–136.7) | 0.796 |
| DIC (%) | 27 (24.5) | 9 (29.0) | 0.602 |
| Pneumonia (%) | 27 (24.5) | 12 (38.7) | 0.109 |
| Severe liver dysfunction(%) | 22 (20.0) | 7 (22.6) | 0.751 |
| Hypoalbuminemia (%) | 28 (25.5) | 11 (35.5) | 0.263 |
| Hypofibrinogenemia (%) | 24 (21.8) | 11 (35.5) | 0.111 |
| Thrombocytopenia (%) | 17 (15.5) | 8 (25.8) | 0.177 |
| Hyponatremia(%) | 56 (50.9) | 20 (64.5) | 0.170 |
| Fever duration(d) | 7.0 (5.0–9.0) | 7.0 (5.0–10.0) | 0.479 |
| Hospital stay(d) | 8.0 (6.0–11.0) | 8.0 (6.0–10.0) | 0.799 |

Figure S2 STROBE-style flow diagram of participant enrollment and analysis cohorts

Supplementary Table S3a. Time effects of immune subsets before and after adjustment for age and sex

|  | β (unadjusted) | FDR *q* | β (adjusted for age + sex) | FDR *q* |
| --- | --- | --- | --- | --- |
| HLA-DR⁺ B cells | 2.21 | <0.001 | 1.67 | 0.026 |
| IgM memory B cells | 1.26 | 0.021 | 0.52 | 0.346 |
| Double-negative B cells | −0.89 | 0.024 | −0.58 | 0.332 |
| Total CD4⁺ T cells | 1.43 | 0.010 | 0.020 | <0.001 |
| Total CD8⁺ T cells | 1.27 | 0.015 | -0.265 | 0.652 |
| Central memory CD8⁺ T cells | 1.58 | 0.015 | 0.507 | 0.196 |
| TEMRA CD8⁺ T cells | 0.62 | 0.040 | 0.166 | 0.603 |
| Total DCs | 1.79 | 0.042 | 0.368 | 0.233 |
| HLA‑DR⁺ monocytes | 2.77 | 0.003 | 0.80 | 0.386 |

Supplementary Table S3b. Group differences in immune subsets between severe and mild cases before and after adjustment for age and sex

|  | β (unadjusted) | FDR *q* | β (adjusted for age + sex) | FDR *q* |
| --- | --- | --- | --- | --- |
| B cell percentage | −17.22 | 0.021 | −15.98 | 0.007 |
| Naive B cells | 20.12 | 0.021 | 19.14 | 0.006 |
| Switched memory B cells | −12.14 | 0.021 | −11.42 | 0.007 |
| Total CD4⁺ T cells | –31.53 | <0.001 | 0.328 | <0.001 |
| Total CD8⁺ T cells | –28.76 | <0.001 | -29.98 | <0.001 |
| Total DCs | -20.37 | 0.042 | 1.04 | 0.798 |
| Total monocytes | 28.96 | 0.001 | -28.98 | <0.001 |

Supplementary Figure S3. Representative model diagnostics for linear mixed‑effects models (CD4⁺ T cells as a representative example). (A) Residuals vs fitted values; (B) Normal Q‑Q plot; (C) Histogram of residuals. The residuals were approximately normally distributed with no substantial heteroscedasticity, supporting the appropriateness of Gaussian linear mixed‑effects models for analyzing proportion data on the original percentage scale.
